# Editorial: Exploring endothelial injury syndromes: mechanisms, markers, and therapeutic potential

**DOI:** 10.3389/fmed.2025.1718272

**Published:** 2025-11-03

**Authors:** Panagiota Anyfanti, Christina Antza, Vasilios Kotsis

**Affiliations:** Third Department of Internal Medicine, Papageorgiou Hospital, Aristotle University of Thessaloniki, Thessaloniki, Greece

**Keywords:** endotheliopathies, endothelial dysfunction, microcirculation, cardiovascular disease, vascular inflammation

The field of endothelial injury syndromes has undergone a significant transformation in recent decades. Initially perceived as a simple anatomical structure, the endothelium is now recognized as a dynamic organ with multifaceted roles in maintaining physiological homeostasis. It is actively involved in regulating coagulation, vascular tone, permeability, and inflammatory responses. When the endothelium is activated, it can lose its integrity, leading to phenotypic changes that trigger procoagulant, pro-inflammatory, and pro-apoptotic mediators (1, 2). This dysfunction is central to a range of hematological conditions and other diseases, including cardiovascular, pulmonary, inflammatory, and tumoral disorders characterized by vascular injury (3–7). Despite the growing attention to endothelial injury syndromes, many questions remain unanswered. There is a need for further research to understand the mechanisms by which endothelial injury contributes to these disorders, develop robust markers for assessing the severity and progression of these syndromes, and explore the endothelium as a potential therapeutic target. Endothelial dysfunction may further serve as a valuable biomarker to guide personalized therapy by identifying patients who are more likely to benefit from targeted interventions.

This Research Topic aimed to explore the multifaceted roles of endothelial injury syndromes in hematology and beyond. Elucidating the mechanisms underlying endothelial injury in various vascular-related disorders will promote the identification and development of endothelial markers for assessing the severity and progression of these syndromes, and ultimately, facilitate the utilization of the endothelium as a therapeutic target. By addressing these objectives, research collected in this Research Topic seeks to advance our understanding of endothelial dysfunction and its implications across different medical specialties. All submitted articles underwent a rigorous peer-review process. Ultimately, ten articles were published.

(i) In the article by Mitiku et al., prevalence and risk factors of deep vein thrombosis were assessed among hospitalized adults in medical and surgical wards, using a conceptual framework of deep vein thrombosis risk stratification, prevalence of deep vein thrombosis, and various clinical factors.
(ii) In the contribution by Xue et al., a case of successful management of late-onset capillary leak syndrome occurring after allogeneic hematopoietic stem cell transplantation was highlighted, employing bevacizumab as a novel therapeutic strategy.
(iii) Alterations in very-low-frequency oscillations were proposed to reflect endothelial dysfunction and contribute to the development of acute kidney injury among neonates with perinatal asphyxia in the research from Botero-Rosas et al., further reinforcing the mechanistic link between renal autoregulation, hemodynamic response, and endothelial dysfunction.
(iv) The role of endothelial dysfunction as a key contributor to the systemic manifestations of chronic obstructive pulmonary disease and a mutual contributor to respiratory diseases and cardiovascular risk was reviewed by Marcuccio et al..
(v) A rare case of intravascular papillary endothelial hyperplasia tumor, provided by Cong et al., further highlights the multifaceted presentation of endothelial injury syndromes.
(vi) In the article by Rudakova et al. presenting a single center's real experience on transplant-associated thrombotic microangiopathy, efficacy of eculizumab, a complement inhibitor, was evaluated as a potential promising therapeutic intervention.
(vii) The role of endothelial glycocalyx in sepsis-induced acute kidney injury was reviewed by Wang et al., with emphasis on the underlying mechanisms linking endothelial glycocalyx with sepsis-induced acute kidney injury and its potential role as a novel therapeutic target for this potentially life-threatening condition.
(viii) Yang et al. constructed a nomogram prediction model with reasonable discriminative capacity for postoperative deep venous thrombosis in arthroplasty.
(ix) In the study by Li et al., serum levels of well-acknowledged markers of endothelial dysfunction, including serum levels of nitric oxide, endothelin-1, and vascular endothelial growth factor, were found to predict post-operative femoral neck fractures after cannulated screw fixation in patients with femoral head necrosis.
(x) In their comprehensive review, Battistoni et al. provided a critical synthesis of clinical and experimental evidence supporting the notion that direct beneficial effects of glucagon-like peptide-1 receptor agonists on endothelial function mediate their cardiovascular protection beyond glycemic control.

This interdisciplinary approach encourages collaboration among basic, translational, and clinical researchers from various specialties, including hematology, cardiology, inflammatory and infectious diseases, nephrology, oncology, and transplantation, to facilitate the exchange of new concepts and update state-of-the-art knowledge regarding mechanisms, markers, and therapeutic potential of endothelial injury syndromes.
